# *Srr2*-dependent SOX2 levels govern the chromatin and transcriptional landscape of adult neural stem cell fate decisions in mouse

**DOI:** 10.1186/s13059-026-04126-7

**Published:** 2026-06-25

**Authors:** Sara Cruces-Salguero, Antonio Jordán-Pla, Ana Domingo-Muelas, Jose Manuel Morante-Redolat, Karine Rizzoti, Ander Matheu, Isabel Fariñas, Robin Lovell-Badge, Veronica Moncho-Amor

**Affiliations:** 1 Cellular Oncology Group, Biogipuzkoa Health Research Institute, Paseo Dr. Beguiristain S/N, San Sebastian, 20014 Spain; 2 Departamento de Biología Celular y Biología Funcional & Instituto de Biotecnología y Biomedicina (BioTecMed), Universidad de Valencia, Burjassot, Spain; 3 Centro de Investigación Biomédica en Red Sobre Enfermedades Neurodegenerativas (CIBERNED), Madrid, Spain; 4 Instituto de Biomedicina de Valencia (IBV-CSIC), Valencia, Spain; 5 Laboratory of Stem Cell Biology and Developmental Genetics, The Francis Crick Institute, 1 Midland Road, London, NW1 1AT UK; 6 IKERBASQUE, Basque Foundation for Science, Bilbao, Spain; 7 Centro de Investigación Biomédica en Red Sobre Fragilidad y Envejecimiento (CIBERFES), Madrid, Spain; 8 Present address: Department of Cell and Developmental Biology, Institute for Regenerative Medicine, Perelman School of Medicine, University of Pennsylvania, Philadelphia, PA USA

## Abstract

**Background:**

Stem cell maintenance and lineage commitment in the nervous system require precise regulation of transcription factors, with SOX2 serving as a pivotal regulator. SOX2 expression is controlled by multiple enhancers, including the *Sox2* regulatory region 2 (*Srr2*). However, the specific role of *Srr2* in adult neurogenesis and chromatin regulation during neural lineage commitment remains incompletely understood.

**Results:**

To dissect the function of *Srr2*, we generate a CRISPR-Cas9 mouse model harboring a targeted deletion of this enhancer. *Srr2* deletion reduce SOX2 levels in proliferating, but not differentiating, neurosphere cultures, while impairing both neuronal and oligodendroglial differentiation. Paired bulk RNA-seq and ATAC-seq during proliferation and early differentiation reveal that loss of *Srr2* induces widespread chromatin compaction during proliferation, which partially converges toward wild-type states upon early differentiation. Multi-omic integration identifies a subset of neurogenic genes exhibiting persistent promoter closure and impaired transcriptional induction in proliferating mutant cells, despite being normally translated during neural differentiation. In vivo, subependymal zone cells of *Srr2*^*del/del*^ mice exhibit lower SOX2 and FOXG1 expression, fewer ASCL1/OLIG2 progenitors, and reduced neuronal and oligodendroglial marker expression.

**Conclusions:**

These findings establish *Srr2* as a critical enhancer of *Sox2* required to maintain a chromatin environment permissive for neural differentiation during stem cell proliferation. Our study underscores the essential role of non-coding regulatory elements in coordinating chromatin accessibility, transcriptional programs, and stem cell fate decisions during adult neurogenesis.

**Supplementary Information:**

The online version contains supplementary material available at 10.1186/s13059-026-04126-7.

## Background

Stem cells (SCs) and their differentiating progeny are hierarchically organized to produce appropriate required cell types during embryonic development and, to a lesser extent, during adult tissue maintenance. In the postnatal mammalian brain, neural stem cells (NSCs) reside in three key neurogenic niches: the subependymal zone (SEZ) in the walls of the lateral ventricles, the subgranular zone (SGZ) of the hippocampal dentate gyrus [1] and the median eminence of the hypothalamus, although the NSCs in the latter tend to only give glial cell types post-puberty [2]. In the SEZ, in particular, NSC divisions give rise to neural progenitor cells (NPCs), which divide a few times before they become early, proliferative, neuroblasts (ENBs). These ENBs migrate anteriorly, subsequently exit the cell cycle as late neuroblasts (LNBs), and ultimately integrate into olfactory bulb circuits as mature inhibitory interneurons [3]. Neurogenesis depends on signals from the microenvironment that activate intrinsic regulators, including transcription factors (TFs) and chromatin remodelers. One remarkable characteristic of subependymal NSCs is their expression of proneural TFs, suggesting that they are already predisposed or primed toward neurogenesis. Adult subependymal NSCs, therefore, exhibit lineage priming with levels of pro-neurogenic fate determinants that are higher than in their embryonic counterparts [4]. This poses the reasonable question of how the balance between stemness and neural fate is molecularly regulated in these NSCs.

SOX2, a member of the *SoxB1* subgroup of TFs, together with SOX1 and SOX3, plays a central role in the regulation of the pluripotency of embryonic/epiblast stem cells (ESCs) [5, 6] and is also essential for a wide variety of processes during embryogenesis [5]. In adulthood, SOX2 exerts functions related to tissue homeostasis and regeneration since it is required for the maintenance of differentiation and self-renewal potential of different somatic SCs [7–9]. SOX2 plays a crucial role in neural development, with a particularly high expression in progenitor cells that decreases as they begin to differentiate [10, 11]. Additionally, SOX2 promotes neuronal differentiation of pluripotent SCs and serves as a key factor in reprogramming astrocytes into neurons [12, 13]. In adults, SOX2 is essential for the self-renewal of NSCs with its expression downregulated during differentiation [14–16]. Moreover, SOX2 is required for oligodendrogenesis and subsequent myelination in the adult brain [17]. SOX2 is, therefore, required for undifferentiated states and for the neural identity, raising questions about its cell-context actions [12, 15].

During differentiation, chromatin undergoes epigenetic remodeling, transitioning from an open, permissive state in SCs to a more compact structure in differentiated cells. For instance, ESCs display more open and active chromatin domains than differentiated cells [18–21], implying a more dynamic chromatin state [22]. Most TFs bind preferentially to open chromatin [22], but pioneer factors like SOX2 can access closed chromatin and initiate its opening process [23]. This activity is intimately tied to SOX2 ability to specifically bind and bend DNA alone or with other proteins such as PAX6, NANOG, OCT4, and BRN2 [24]. SOX2 binds to both pluripotency genes and neural fate genes in a dosage-dependent manner [25], allowing neural induction when pluripotency signals are lost through its chromatin-opening action in specific neural genes [26].

The transcriptional regulation of SOX2 is complex. The expression of *Sox2* in ESCs and in fetal NPCs is mainly regulated by a group of intragenic regulatory regions: *Srr1*, *Srr2* and *Srr18*, *Srr107,* and *Srr111* [27–29]*. Sox2* regulatory region 2 (*Srr2*), in particular, is an enhancer located 4 kb downstream of the transcription start site and the main regulator of *Sox2* expression in SCs [30]. *Srr2* contains a SOX2-binding motif that is bound by OCT3/4-SOX2 or OCT6/SOX2 heterodimers in ESCs [27]. Furthermore, it has been shown to enhance *Sox2* expression in ESCs, the fetal telencephalon [27], and some adult tissues [29]. In glioblastoma, *Srr2* deletion suppresses *Sox2* expression and reduces its oncogenic activity [31]. We have previously shown that cell cycle exit and differentiation of adult subependymal NSCs/NPCs involve repression of *Sox2* expression by cyclin-dependent kinase inhibitor p27 and co-repressors, both at its promoter and *Srr2* enhancer, but the specific role of *Srr2* in adult NSC/NPC differentiation remains elusive [15].

In this study, we used a mouse model with a targeted *Srr2* deletion [32], along with a well-established in vitro system of adult NSC/NPC differentiation [15, 33], to dissect the role of *Srr2* in *Sox2* regulation, and its impact on adult NSC properties. We combined transcriptomic, chromatin accessibility, and DNA–protein interaction analyses to explore how *Srr2* influences SOX2-driven gene expression programs. Altogether, our findings reveal that *Srr2* is essential for precise modulation of SOX2 levels and, in turn, for orchestrating chromatin dynamics during cell fate transitions. Specifically, the levels of SOX2 are important to maintain an open chromatin state required for proper differentiation into oligodendroglial and neuronal lineages.

## Results

### The Srr2 enhancer controls Sox2 expression in adult NSCs and ensures proper lineage progression

In adult neurogenic niches, SOX2 labels a heterogeneous population that includes both NSCs and NPCs, and its expression becomes sharply reduced in committed NBs that exit the cell cycle [12, 15, 16, 29, 34–36]. Interestingly, SOX2 effects are dosage and cell context-dependent [12, 37, 38], but the regulation of *Sox2* levels at the transcriptional level has not been completely deciphered. To assess whether *Srr2* modulates *Sox2* expression in adult neurogenic niches, we used a mouse line carrying a CRISPR-Cas9-mediated deletion of the *Srr2* enhancer [32] and we derived cultures from the SEZ of 2-month-old wild-type (WT) and *Srr2*^*del/del*^ mice [33]. Under non-adhesive conditions in the presence of mitogens bFGF and EGF, proliferative NSCs isolated from the SEZ grow as floating neurospheres that can be propagated for a number of passages [33]. Under these proliferative conditions, *Srr2*^*del/del*^ neural stem/progenitor cells could be maintained over time and efficiently propagated as neurosphere cultures despite showing lower levels of *Sox2* (Additional file 1: Fig. S1A-D).

**Fig. 1:**
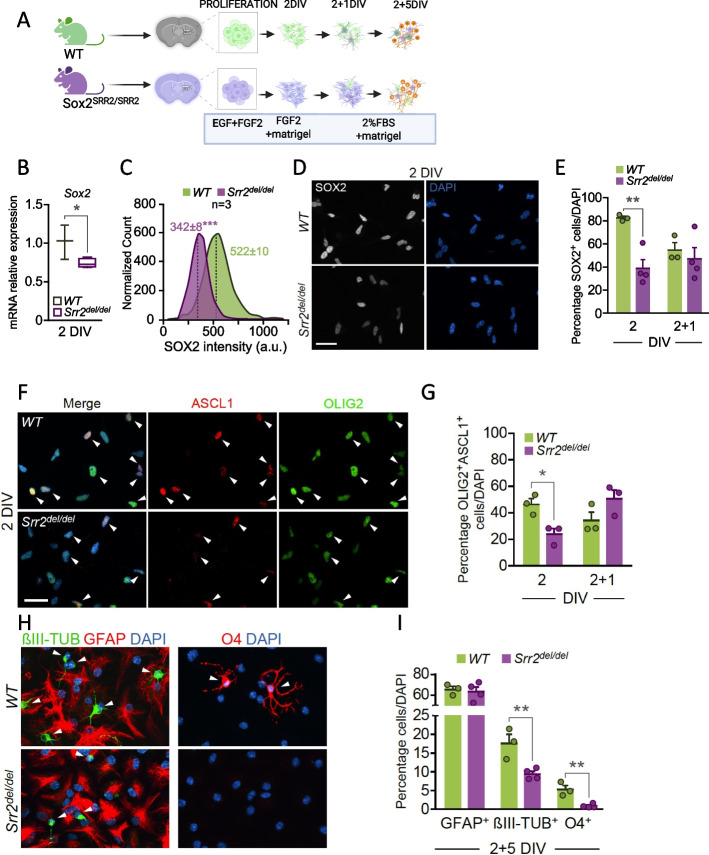
*Srr2* enhancer regulates *Sox2* expression and lineage progression in adult NSCs in vitro. **A** Schematic drawing of the NSC differentiation protocol, DIV indicates days in vitro. **B** *Sox2* expression by RT-qPCR at 2 DIV in 2-mo-old *Srr2*^*del/del*^ and WT. **C** SOX2 expression by IF at 2 DIV in 2-mo-old *Srr2*^*del/del*^ and WT. Cells were classified as SOX2^high^ using a threshold of 400 arbitrary units (a.u.) **D** Representative immunocytochemistry images for SOX2 at 2 DIV cultures from 2-mo-old *Srr2*^*del/del*^ and WT SEZ. **E** Percentage of SOX2^high^ cells at 2 and 2 + 1 DIV cultures from 2-mo-old *Srr2*^*del/del*^ and WT SEZ. **F** Representative immunocytochemistry images for OLIG2 + ASCL1 + cells at 2 DIV cultures from 2-mo-old *Srr2*^*del/del*^ and WT SEZ. **G** Percentage of OLIG2 + ASCL1 + cells at 2 and 2 + 1 DIV cultures from 2-mo-old *Srr2*^*del/del*^ and WT SEZ. **H** Representative immunocytochemistry images for GFAP +, βIII-TUB +, O4 + cells at 2 + 5 DIV cultures from 2-mo-old Srr2del/del and WT SEZ. **I** Percentage of GFAP +, βIII-TUB +, O4 + cells at 2 + 5 DIV cultures from 2-mo-old *Srr2*^*del/del*^ and WT SEZ. Data are presented as mean ± SEM from three independent cultures per genotype (*n* = 3 WT and n = 3 *Srr2*.^*del/del*^ cultures)

Neurosphere cells can be induced to differentiate by stepwise changes in culture conditions [33]. We next addressed whether the transition from proliferation to cell cycle arrest or the differentiation process were impaired after *Srr2* deletion. Neurosphere cells of both genotypes were dissociated and single cells were seeded onto Matrigel with bFGF for 2 days in vitro (2 DIV; PROLIF); then the medium was changed to mitogen-free medium to allow the cells to stop dividing and start differentiation (DIFF) and cultures were analyzed after 1 or 5 days of differentiation (2 + 1 DIV and 2 + 5 DIV, respectively; Fig. 1A). At 2 DIV, *Srr2*-deficient proliferating NSC/NPCs exhibited significantly reduced *Sox2* mRNA and protein levels (Fig. 1B, C), resulting in a lower proportion of SOX2^high^ cells in the cultures (Fig. 1D, E). By 2 + 1 DIV at DIFF, however, SOX2 levels were comparable between genotypes (Fig. 1E). This is consistent with our previous observation that *Srr2* is repressed by p27 at the onset of differentiation and, therefore, *Srr2* deletion at this time does not influence *Sox2* expression. These findings indicate that *Srr2* is essential for maintaining *Sox2* expression during the proliferative phase of NSCs/NPCs.

We previously reported that SOX2 plays a critical role in positively regulating the expression of TFs ASCL1 and OLIG2 during NSC/NPCs differentiation [15]. In line with this, *Srr2*-deficient cultures expressing reduced levels of SOX2 also showed decreased proportions of NPCs expressing these TFs during their proliferative phase, but not at 2 + 1 DIV during early DIFF (Fig. 1F, G). This premature reduction in OLIG2 and ASCL1 levels in an actively expanding NPC population subsequently resulted in a significant decline in oligodendrocyte and neuron numbers in fully differentiated cultures at 2 + 5 DIV (Fig. 1H, I). These findings are consistent with earlier reports that neurons derived from *Sox2* hypomorphic NSCs fail to mature unless *Sox2* is restored early in culture [37]. To rule out a potential effect of *Sox2* reduction on NPC cell cycling, we assessed proliferation by both Ki67 immunostaining and EdU incorporation. This analysis revealed a strong reduction in proliferative activity at the onset of differentiation in both WT and *Srr2*^*del/del*^ cultures, with minimal EdU incorporation in either genotype, indicating cell-cycle exit upon differentiation (Additional file 1: Fig. S1E). In addition, DAPI quantification revealed no significant differences in total cell numbers between genotypes at this stage, arguing against increased cell loss (Additional file 1: Fig. S1F). Given that equal numbers of cells were plated at the onset of differentiation, and together with the comparable cell-cycle exit observed by Ki67 and EdU analyses, these data support the conclusion that *Srr2*-dependent SOX2 levels primarily impact lineage differentiation rather than cell proliferation or cell survival.

### Srr2 regulates chromatin accessibility and gene expression programs essential for early neural lineage progression

**Fig. 2:**
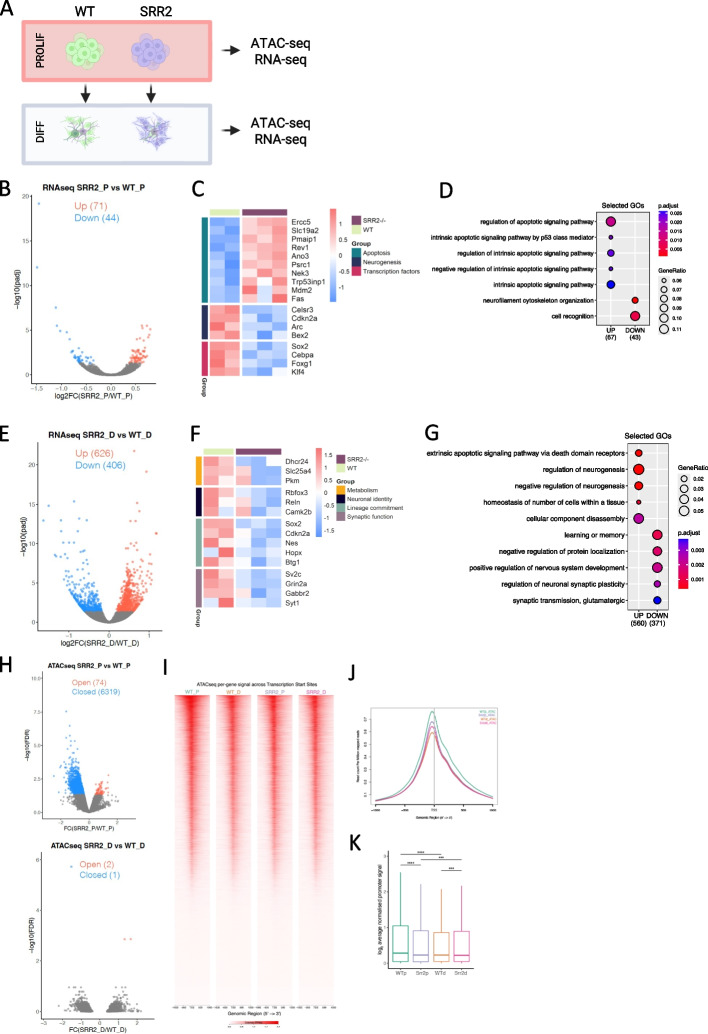
*Srr2* regulates chromatin accessibility and transcriptional programs essential for early neural lineage progression in vitro. **A** Schematic drawing of the omics data and experimental sampling points used in this study. **B** Volcano plot of the DEGs at 2 DIV in 2-mo-old *Srr2*^*del/del*^ and WT. Red dots represent up-regulated genes, and blue dots down-regulated genes. Threshold was established at adjusted *p*-value < 0.05. **C** Heatmap showing DEGs from enriched pathways at 2 DIV (2-mo-old *Srr2*^*del/del*^* vs* WT). Expression values are z-scored; blue represents lower and red higher expression across conditions. **D** Dot plot of enriched pathways for DEGs at 2 DIV in the *Srr2*^*del/del*^* vs* WT comparison. Pathway enrichment was performed in MetaCore. **E** Volcano plot of DEGs at 2 + 1 DIV from 2-mo-old *Srr2*^*del/del*^ and WT samples. Red dots indicate upregulated genes and blue dots indicate downregulated genes. The significance threshold was an adjusted p-value < 0.05. **F** Heatmap of DEGs within enriched pathways at 2 + 1 DIV in 2-mo-old *Srr2*^*del/del*^ and WT. Expression values are z-scored; blue represents lower and red higher expression across conditions. **G** Dot plot of pathways enriched among DEGs at 2 + 1 DIV in the *Srr2*^*del/del*^ vs WT comparison. Enrichment was performed using MetaCore. **H** Volcano plots of differentially accessible chromatin peaks at 2 DIV (top) and 2 + 1 DIV (bottom) in 2-mo-old *Srr2*^*del/del*^ vs WT. Each dot represents an ATAC-seq peak; significant peaks indicate increased or decreased accessibility between genotypes at the indicated time point. **I** Clustered heatmaps of ATAC-seq in 2 DIV (P) and 2 + 1 DIV (D) *Srr2*^*del/del*^ vs WT. **J** Average ATAC-seq signal profiles across all transcription start sites (± 1 kb). Notably, the accessibility profile of proliferating *Srr2*^*del/del*^ cells (2 DIV) closely resembles that of differentiating WT cells (2 + 1 DIV), whereas WT proliferating cells (WT_P) display a distinctly higher accessibility across promoter regions. **K** Boxplots represent the distribution of ATAC-seq signal intensity at promoter regions for each condition. Median accessibility values are indicated: WT_P = 0.349, WT_D = 0.250, *Srr2*_P = 0.283, *Srr2*_D = 0.255. P-value: paired two-tailed Student’s t test. Boxplots show median values and first to third interquartile ranges. RNA-seq analyses were performed using independent biological replicates (*n* = 2 WT and *n* = 3 *Srr2*^*del/del*^ cultures). ATAC-seq analyses were performed using three independent biological replicates per genotype (*n* = 3 WT and *n* = 3 *Srr2*.^*del/del*^ cultures)

To investigate the effects of the *Srr2* enhancer deletion on gene expression programs during NSC proliferation and early lineage commitment, we performed both paired bulk RNA sequencing (RNA-seq) and chromatin accessibility analysis (ATAC-seq) on subependymal cultures during PROLIF and 2 + 1 DIV (DIFF) conditions. The latter corresponds to an early commitment window, when cells exit cycle and initiate proneural/oligodendroglial programs but before late differentiation markers and cell-type heterogeneity accumulate (Fig. 2A). At the PROLIF stage, we identified 115 differentially expressed genes (DEGs) between *Srr2*-deficient and WT samples (Fig. 2B), indicating an early transcriptional divergence driven by the absence of this enhancer. Among them, 71 genes were upregulated in *Srr2*^*del/del*^ samples, including key apoptosis-related genes such as *Pmaip1, Trp53inp1, Mdm2*, *Fas*, *Psrc1, Ercc5, Nek3, Ano3, Slc19a2,* and *Rev1.* Conversely, 44 genes were downregulated, including neurogenesis-associated *Foxg1, Arc, Cdkn2a, Cel*sr3, and *Bex2,* and self-renewal-associated transcription factors *Cebpa, Foxg1, Klf4,* and *Sox2* itself [39–41] (Fig. 2C). Enrichment analysis using Gene Ontology (GO) revealed statistically significant changes in biological processes involved in apoptosis, neurofilament cytoskeleton, cell recognition, and adhesion molecules in *Srr2*^*del/del*^ cells (Fig. 2D and Additional file 2: Table S1-S2).

At the onset of differentiation, RNA-seq analyses on 2 + 1 DIFF cultures led to the characterisation of 1,032 DEGs, with 626 up-regulated and 406 down-regulated in the *Srr2*^*del/del*^ compared to WT cells (Fig. 2E). Notably, several genes critical for SC maintenance and lineage commitment were downregulated, including *Sox2, Nes, Cdkn2a, Hopx,* and *Btg1*. Genes associated with neuronal identity, such as *Rbfox3 (NeuN), Reln,* and *Camk2b*, also showed reduced expression. In addition, key metabolic genes relevant to neuronal function as *Slc25a4, Pkm,* and *Dhcr24* were downregulated, along with genes involved in synaptic function, including *Syt1, Grin2a, Sv2c, Reln,* and *Gabbr2* (Fig. 2F). Enrichment analysis using GO revealed many processes affected by *Srr2* deletion, including regulation of neurogenesis, regulation of cell cycle, and regulation of neuronal synaptic plasticity (Fig. 2G and Additional file 2: Table S3-S4). These results suggested a coordinated transcriptional reduction of genes essential for the establishment, maintenance, and functional maturation of the neuronal lineage.

Given our previous work on the regulatory relationship between p27 and *Sox2* [15], we next asked whether the reduction in *Sox2* dosage induced by *Srr2* deletion could elicit feedback effects on p27 expression or its associated transcriptional programs. Analysis of our RNA-seq datasets revealed no significant changes in *Cdkn1b* transcript levels between WT and *Srr2*^*del/del*^ NSCs, either under 2 DIV PROLIF or 2 + 1 DIFF (data not shown). Consistent with this, cross-referencing our differentially expressed genes with a published catalogue of p27-associated targets [42] showed only minimal overlap and no significant enrichment in any of the conditions analyzed (Additional file 1: Fig. S1G). Together, these results suggest that the decrease in SOX2 levels resulting from *Srr2* deletion does not lead to detectable transcriptional feedback on p27, nor does it substantially impact p27-associated gene networks under the conditions examined.

As a pioneer transcription factor, SOX2 has been shown to prime chromatin for the activation of downstream transcriptional programs [43]. To investigate the regulatory landscape shaped by the *Srr2* enhancer during NSC proliferation and early lineage commitment, we performed paired chromatin accessibility profiling (ATAC-seq) on the same cultures at PROLIF and DIFF conditions. During PROLIF, we identified 6,393 differentially accessible regions (DARs) between WT and *Srr2*^*del/del*^ samples, of which 6,319 regions were less accessible in the mutant, indicating widespread chromatin compaction in the absence of *Srr2* (Fig. 2H, upper and Additional file 2: Table S5). In contrast, only 3 DARs were detected at 2 + 1 DIFF, suggesting a convergence of chromatin states at this stage (Fig. 2H, bottom and Additional file 2: Table S6). Strikingly, measuring ATAC signal around Transcription Start Sites (TSS) showed that the chromatin accessibility profile of *Srr2*^*del/del*^ cells during proliferation resembles that of WT cells undergoing differentiation, implying a premature chromatin compaction (Fig. 2I, J). Furthermore, *Srr2*^*del/del*^ cells failed to undergo the chromatin closing transition normally observed in WT cells during early differentiation (Fig. 2I, J), and they exhibited a globally reduced promoter signal compared to WT controls in proliferation (Fig. 2K). The results indicated that *Srr2*-dependent maintenance of sufficient levels of SOX2 appears to be required to sustain an open chromatin state during proliferation.

### SOX2-dependent chromatin priming during proliferation enables timely activation of proneural programs at neurogenic onset

**Fig. 3:**
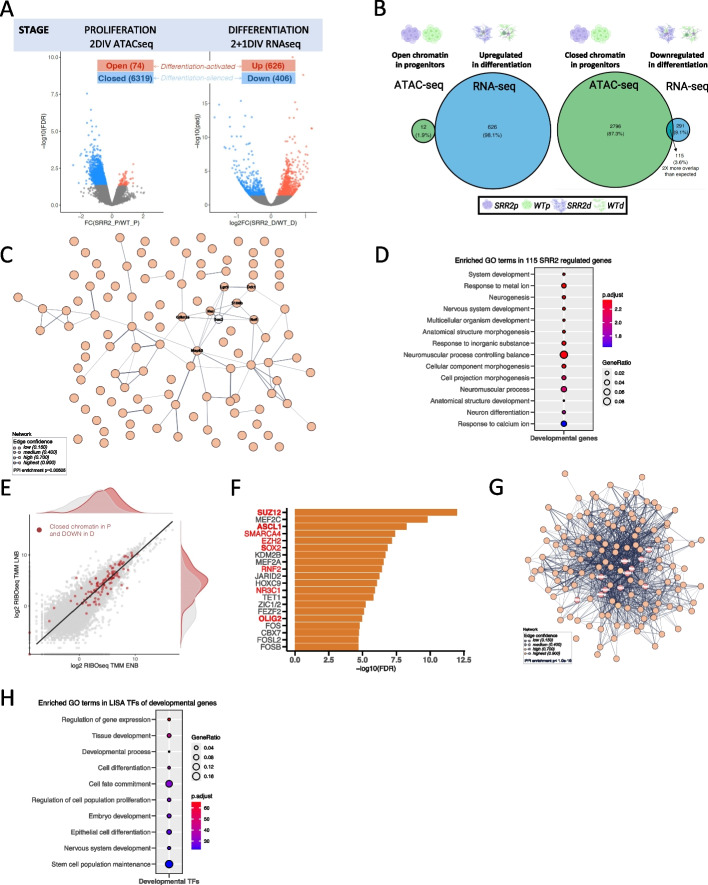
SOX2 controls chromatin accessibility in proliferating NSCs to enable timely neurogenic commitment. **A** Schematic hypothesis: in proliferating NSCs, open chromatin regions should map to genes that become upregulated at differentiation (DIFF), whereas closed regions should map to genes that become transcriptionally repressed. **B** Venn diagram comparing genes associated with open chromatin in proliferation (ATAC-seq) and genes upregulated during early differentiation (DIFF): no significant overlap (left panel) and Venn diagram comparing genes associated with regions that remain closed in *Srr2*^*del/del*^ during proliferation and genes downregulated at DIFF: ~ twofold higher overlap than expected (right panel). **C** Protein–protein interaction network generated from 115 genes showing reduced transcriptional induction during early differentiation in mutant cells (STRING PPI enrichment *p* = 0.00505). Line thickness indicates the strength and confidence of the connection (https://string-db.org/). **D** Representative Gene Ontology (GO, Biological Process) terms enriched in the subset of 115 genes repressed at the transcriptional level during early differentiation in mutant cells. Significance values are reported after FDR correction. **E** Scatterplot comparing the 115 genes repressed in mutants with translation efficiency from subependymal neuroblasts (NBs) at early (ENB) and late (LNB) stages, using published Ribo/RNA-seq data [46]. **F** Bar plot of enrichment scores for the top transcription factors and chromatin regulators associated with genes specifically translated at ENB/LNB. Scores were obtained with LISA (http://lisa.cistrome.org). **G** Protein–protein interaction network generated from 140 putative candidate TFs and chromatin regulators. Line thickness indicates the strength and confidence of the connection (https://string-db.org/). Labeled in red proteins involved in Signaling pathways regulating pluripotency of stem cells. **H** Representative Gene Ontology (GO, Biological Process) terms enriched in LISA TF. Significance values are reported after FDR correction

One characteristic of subependymal NSCs is their lineage priming, a predisposition toward neurogenesis characterized by the expression of proneural TFs [4], a feature that is also observed in NSCs during early neurogenesis [44, 45]. Because our data suggested that normal levels of SOX2 appear necessary to maintain chromatin accessibility during the proliferative phase, we hypothesized that this accessibility might facilitate the expression of proneural genes upon differentiation onset. Accordingly, we asked whether transcriptional changes observed during early differentiation could be linked to chromatin accessibility states established during proliferation. To test this hypothesis, we examined whether open chromatin regions at 2 DIV PROLIF were associated with genes transcriptionally upregulated at 2 + 1 DIV DIFF and closed chromatin regions corresponded to genes that would become transcriptionally repressed (Fig. 3A). The analysis revealed that genes with open chromatin during proliferation did not significantly overlap with genes upregulated during early differentiation (Fig. 3B). In contrast, genes associated with regions that remained closed in *Srr2*^*del/del*^ cells during proliferation showed a two-fold higher overlap than expected with genes that were downregulated at DIFF (Fig. 3B). To assess whether these genes represent a coordinated biological program rather than isolated transcriptional changes, we performed GO enrichment and protein–protein interaction network analyses. Notably, we identified a subset of 115 genes with reduced induction at the transcriptional level during early differentiation in mutant cells that cluster into a significantly enriched protein–protein interaction (PPI) network according to STRING (*p* = 0.005) (Fig. 3C, Additional file 3: Fig. S1). GO enrichment analysis of these 115 genes with reduced induction revealed strong associations with biological processes such as neurogenesis, nervous system development, and neuron differentiation (Fig. 3D and Additional file 2: Table S7). These findings suggested that SOX2 contributes to the epigenetic priming of adult NSCs for neurogenesis by modulating chromatin accessibility during the proliferative phase. In particular, reduced SOX2 activity is associated with decreased chromatin accessibility at early neurogenic loci, correlating with impaired induction of genes required for early neuronal differentiation. This highlights a role for SOX2 not only in maintaining NSC identity but also in preparing the chromatin landscape to ensure timely activation of proneural programs at the onset of differentiation.

Because transcriptional repression in mutants could be particularly consequential if the affected genes were those highly translated during neurogenesis, we queried published sequencing data of NBs ribosome-bound mRNAs obtained from RiboTag mice, in which the endogenous coding sequence for the ribosomal large subunit protein RPL22 was replaced by a Cre-recombinase-dependent HA-tagged variant, crossed to Doublecortin (*Dcx*)-Cre recombinase transgenic mice for recombination in NBs [46]. Deep sequencing of the total RNA (transcriptome) and of the HA-immunoprecipitated ribosome-bound RNA (translatome) in these mice had revealed an uncoupling between transcription and translation in NBs [47]. We compared our set of 115 repressed genes in *Srr2*^*del/del*^ cells with the Ribo-seq/RNA-seq data from subependymal ENBs and LNBs. Our analysis revealed that these 115 genes are more translated on average than the rest of the mRNAs in both ENBs and LNBs (Fig. 3E).

To identify upstream regulators potentially responsible for the activation of these genes specifically translated at ENB and LNB, we next applied LISA to infer upstream regulators most likely driving the observed transcriptional and chromatin changes (http://lisa.cistrome.org), which predicted 140 putative candidate TFs and chromatin regulators. Among the 20 most statistically significant regulators, prominent candidates included SOX2, SUZ12, OLIG2, FOS, CBX7, FOSL2, and FOSB. Taken together, this gene set captures the balance between chromatin repression and activation during neural fate decisions: PRC2/PRC1 components (SUZ12, EZH2, JARID2, RNF2, CBX7, KDM2B) enforce H3K27 and H2A-ubiquitin–mediated silencing, whereas SOX2, SMARCA4 (BAF), and TET1, together with activity- and context-responsive regulators (AP-1: FOS/FOSL2/FOSB; MEF2A/MEF2C; NR3C1) and lineage TFs (ASCL1, OLIG2, FEZF2, HOXC9, ZIC), promote enhancer accessibility and transcriptional programs that specify and mature neural lineages (Fig. 3F).

To evaluate whether these candidate regulators form coherent functional modules, we performed a PPI network analysis (STRING) of the full set of 140 LISA-inferred candidates. Notably, we found an enrichment in proteins involved in the KEGG pathway “Signaling pathways regulating pluripotency of stem cells” labeled in red in the STRING (Fig. 3G, Additional file 3: Fig. S2). Additionally, GO enrichment analysis of these 140 putative candidate TFs and chromatin regulators revealed strong associations with biological processes such as regulation of gene expression, tissue development, cell differentiation, cell fate commitment, nervous system development, and maintenance of SC populations (Fig. 3H and Additional file 2: Table S8). Together, these findings support a model in which SOX2-dependent chromatin priming during the proliferative phase enables timely transcriptional activation of proneural gene programs during early stages of differentiation that are specifically translated during NB commitment and differentiation.

### Srr2 is required in vivo to maintain nsc identity and support proper neuronal and oligodendroglial differentiation in the SEZ

**Fig. 4:**
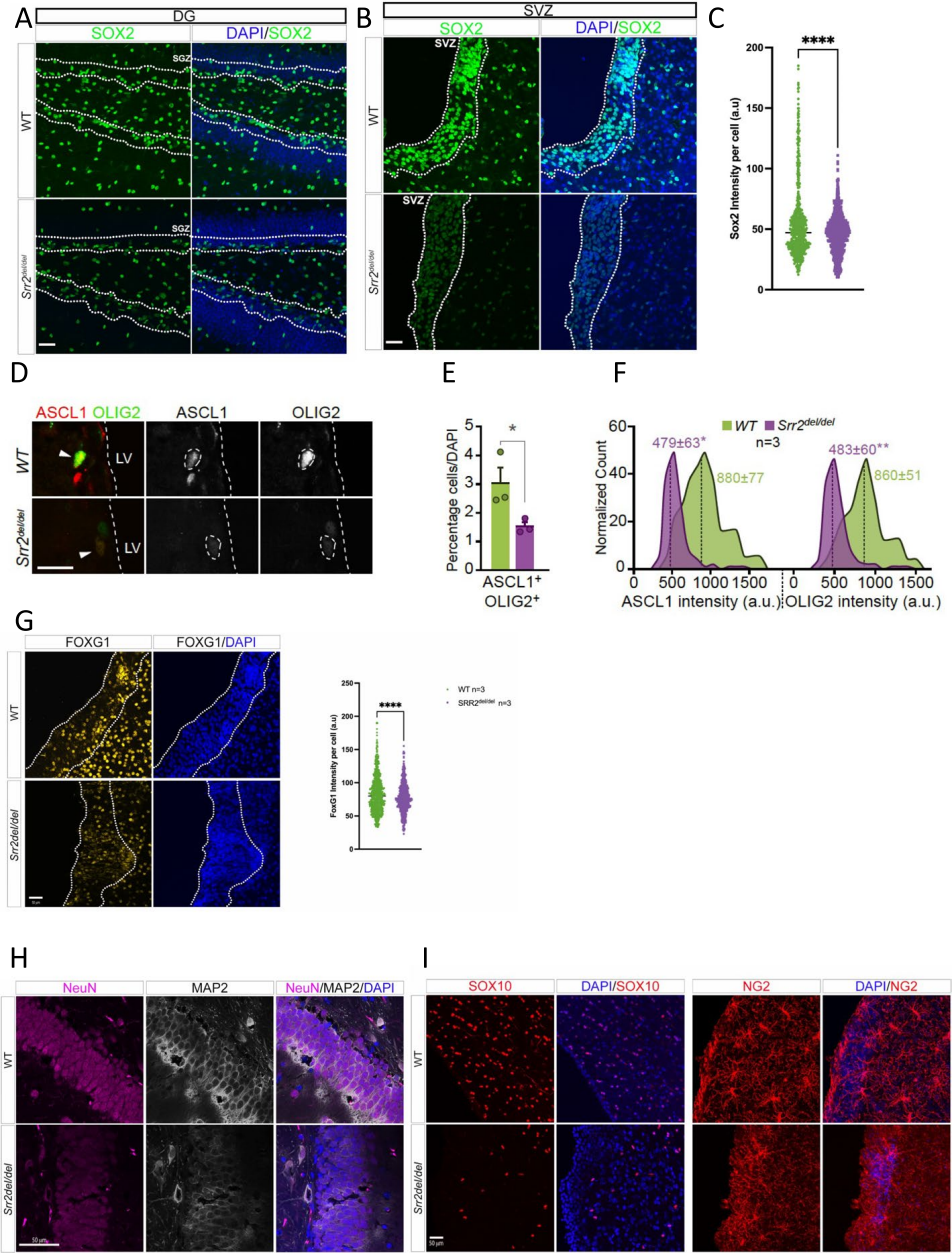
*Srr2* is required in vivo to preserve SOX2 expression, NSC identity, and balanced neuronal and oligodendroglial differentiation in the SEZ. **A**, **B** Immunofluorescence of SOX2 in *Srr2*^*del/del*^* vs* wild type (WT) at 2-mo-old. Regions of interest corresponding to the lateral ventricle/SEZ (**A**) and dentate gyrus (DG) (**B**) were delineated on anatomically matched sections at equivalent rostro–caudal levels for all genotypes. **C** Quantification of SOX2 expression by immunofluorescence in the SEZ (*Srr2*^*del/del*^* vs* WT). Bars show the mean staining intensity of SOX2⁺ cells. **D** Representative immunofluorescence images for ASCL1 (red) and OLIG2 (green) in the SEZ of WT and *Srr2*^*del/del*^ mice. **E** Percentage of ASCL1 + OLIG2 + double positive cells in the SEZ of 2-mo-old WT and *Srr2*^*del/del*^ mice. **F** Quantification of ASCL1 and OLIG2 expression intensity in cells in the SEZ of WT and *Srr2*^*del/del*^. **G**-**J** Representative immunofluorescence images from 2-mo-old *Srr2*^*del/del*^ and WT mice: **G** Immunofluorescence for SOX2 (green), FOXG1 (red), and DAPI (blue) in the subventricular zone (SEZ) of 2-mo-old WT and *Srr2*^*del/del*^ mice. Dashed lines outline the SEZ region (left panel). Scale bar, 50 μm. Right panel shows quantification of FOXG1 fluorescence intensity in SEZ cells from WT and *Srr2*^*del/del*^ mice. **H** Neurogenesis markers NeuN and MAP2 in the SEZ. **J** Oligodendrogenesis markers SOX10 and NG2 in the SEZ. Data are presented as mean ± SEM from three independent cultures per genotype (*n* = 3 WT and *n* = 3 *Srr2*^*del/del*^ cultures)

Our data indicated that *Srr2* sustains a level of SOX2 during proliferation required to maintain an active chromatin state in genes that are required during differentiation and are translated at the NB stage. Having established a mechanistic role for *Srr2* in regulating *Sox2* and subsequent chromatin accessibility in vitro, we next investigated whether *Srr2*^*del/del*^ mice exhibit impaired neurogenesis. We examined the gross morphology of the SEZ and the SGZ, both of which appeared structurally intact in mutant and control animals. However, immunofluorescence analysis revealed a marked reduction in SOX2 levels in both *Srr2*^*del/del*^ neurogenic niches at 2 months of age, while the proportion of SOX2 positive cells was not significantly altered (Fig. 4A, B and Additional file 1: Fig. S2A). Quantitative analysis at the single-cell level in the SEZ further confirmed a measurable significant decrease in SOX2 protein levels (Fig. 4C). Consistent with the preserved gross morphology of the SEZ and SGZ, TUNEL and CC3 immunostaining did not reveal genotype-dependent differences in apoptotic cells in the SEZ or DG (Additional file 1: Fig. S2B), supporting that the in vivo changes primarily reflect altered transcription factor levels and lineage-associated populations rather than increased cell death.

We had previously demonstrated that repression of *Sox2* by the cyclin-dependent kinase inhibitor p27 leads to reduced numbers of ASCL1^+^/OLIG2^+^ NPCs in the SEZ [15]. In line with these data and our in vitro data (see Fig. 1), we confirmed that *Srr2*-deficient mice also showed a significant reduction in the numbers of ASCL1^+^/OLIG2^+^ NPCs, accompanied by decreased immunofluorescence intensity for both TFs (Fig. 4D–F). Moreover, FOXG1, a key TF that contributes to chromatin remodeling and cooperates with SOX2 and OLIG2 to maintain NSC identity and prevent premature differentiation, was also downregulated in the SEZ of *Srr2*^*del/del*^ mice compared to WTs (Fig. 4G and Additional file 1: Fig. S2C).

To further assess lineage commitment in vivo, we examined the expression of neuronal and oligodendroglial general markers. Immunostaining revealed reduced levels of the neuronal markers MAP2 and NeuN in the DG of *Srr2*^*del/del*^ mice (Fig. 4H), alongside lower levels of oligodendroglial markers SOX10 and NG2 in the SEZ (Fig. 4I and Additional file 1: Fig. S2D). These results suggested that loss of the *Srr2* enhancer impairs both neuronal and oligodendrocyte lineage progression in the adult SEZ.

## Discussion

Our findings uncover a crucial role for the *Srr2* enhancer in sustaining *Sox2* expression and coordinating chromatin accessibility programs that ensure proper lineage progression during adult neurogenesis. Previous studies have demonstrated that deletion of a broader *Sox2* regulatory region encompassing *Srr2* (SRR2–18) in embryonic stem cells impairs *Sox2* expression, chromatin accessibility, and neural differentiation of ESC-derived neural stem/progenitor cells [48]. In our study, by deleting *Srr2*, a well-characterized *Sox2* enhancer active in SC/progenitor contexts [27–29], and combining in vivo and in vitro analyses with paired RNA-seq and ATAC-seq at defined stages, we show that *Srr2* is required to sustain SOX2 levels during proliferation, preserve accessibility at neurogenic regulatory elements, and enable timely activation of proneural and oligodendroglial programs upon early differentiation.

SOX2 has long been recognized as a key regulator of NSC identity and multipotency [12, 16, 38], yet the mechanisms that maintain its precise dosage in the adult brain have remained unclear. Our data identify *Srr2* as a cis-regulatory element required to preserve *Sox2* expression specifically during proliferation [27, 29]. The temporal specificity of *Srr2* function, active in proliferating NSCs but silenced upon differentiation, suggests a dual mechanism in which *Sox2* levels are first maintained by enhancer-driven transcription and later down-regulated through p27-dependent repression [41, 49]. Consistent with this regulatory hierarchy, *Cdkn1b* (p27) expression was not altered in *Srr2*^*del/del*^ cells and differentially expressed genes did not show enrichment for previously reported p27-associated targets [42]. This enhancer-mediated control of *Sox2* ensures the balance between self-renewal and differentiation competence [50].

The combined RNA-seq and ATAC-seq analyses reveal that loss of *Srr2* disrupts both transcriptional and epigenetic landscapes with distinct temporal dynamics. During proliferation, *Srr2*^*del/del*^ cells exhibit widespread loss of accessibility together with downregulation of genes associated with neurogenesis, self-renewal, and neuronal metabolism and activation of apoptotic pathways. Although apoptosis and stress related GO terms were enriched, we did not detect increased cell loss or apoptosis, as cell numbers remained comparable in vitro and TUNEL/cleaved Caspase-3 staining showed no genotype differences in vivo. Stress and apoptosis-related GO term enrichments may be reflecting a transcriptional stress-response signature when SOX2 levels are reduced. Importantly, the chromatin accessibility profile of *Srr2*^*del/del*^ cells during proliferation resembles that of WT cells entering differentiation, suggesting that insufficient SOX2 levels drive a premature transition toward a more restrictive chromatin configuration. Thus, *Srr2*-dependent *Sox2* expression maintains an open and transcriptionally permissive chromatin state necessary for NSC competence [18].

At early differentiation, global accessibility largely converges between genotypes, yet a strong transcriptional divergence emerges. This temporal offset indicates that *Srr2*-dependent SOX2 acts pre-commitment to establish open chromatin that lineage factors subsequently read out [23, 43, 47, 51]. If this threshold is not achieved, chromatin prematurely compacts and the transcriptional response to differentiation cues is attenuated, consistent with SOX2’s dosage sensitivity and pioneer-like properties [8, 9, 12, 23, 25, 51].

Promoters that remain closed in *Srr2*^*del/del*^ cells during proliferation are overrepresented among genes that fail to up-regulate upon early differentiation, yielding a core set of 115 genes enriched for regulators of neurogenesis and neuronal function. Regulator-inference analysis identifies SOX2, ASCL1, and OLIG2, together with Polycomb complex components (SUZ12, EZH2, JARID2, RNF2, CBX7, and KDM2B), as upstream influences on the misprogrammed 115 gene set. This supports a model in which SOX2 acts as a pioneer factor opposing Polycomb-mediated repression to preserve baseline chromatin accessibility and RNA polymerase II competence at lineage-relevant loci [52]. In *Srr2*^*del/del*^ cells, insufficient SOX2 may leave these sites unprotected, allowing default repressive mechanisms to dominate and shifting the system toward an epigenetic state that resembles the wild-type differentiation pattern even before commitment begins. Importantly, the reduced expression of this 115-gene set is best interpreted as a failure of timely transcriptional induction rather than evidence of active transcriptional repression. As our conclusions are based on differential expression following enhancer deletion, the observed downregulation likely reflects insufficient activation downstream of reduced SOX2 levels, rather than the direct repressive mechanisms. Nevertheless, failure to establish SOX2-dependent chromatin priming during proliferation may render these loci more permissive to repressive processes, including Polycomb-mediated chromatin regulation, which could further stabilize a transcriptionally less competent state during early differentiation.

Our time window approach was designed to capture the earliest transcriptional effects of enhancer loss while minimizing secondary feedback. At this stage, accessibility differences diminish whereas transcriptional divergence increases, indicating that enhancer-dependent priming leaves a lasting transcriptional signature even after global chromatin states converge, consistent with chromatin remodeling acting upstream of transcriptional activation [6, 12, 22, 41]. This decoupling between accessibility and expression aligns with previous studies of the SOX2 network [37]. Furthermore, the 115 downregulated genes are enriched among mRNAs preferentially translated during neuroblast stages, suggesting that *Srr2*-dependent priming during proliferation influences later translational checkpoints [53]. We propose that enhancer-tuned SOX2 helps establish promoter architectures (e.g., paused polymerase and accessible core motifs) that ease recruitment of lineage TFs and cofactors. Failure to install this architecture results in delayed or inefficient transcript accumulation, preventing proper synchronization with neuroblast-stage translational programs [25, 26, 47].

Although the in vivo phenotype in mice is modest, this likely reflects functional redundancy within the *Sox2* regulatory architecture (multiple enhancers) [27, 29] and partial compensation by SOX3 [54], in contrast to the in vitro system, where an isolated context and a defined time window more stringently expose enhancer dependence. Even so, the in vivo data align with the in vitro model: *Srr2* deletion lowers SOX2 levels and reduces ASCL1⁺/OLIG2⁺ progenitor intermediates that link stemness with neuronal and oligodendroglial lineages [10, 13, 15, 17, 43, 55]. The reduction of FOXG1 provides an additional axis by which SOX2 dosage may influence competence, given FOXG1’s roles in chromatin remodeling, suppression of premature differentiation, and cooperation with SOX2 to enforce neural stem identity [39]. Consequently, neuronal and oligodendroglial output is reduced, although the overall architecture of the neurogenic niches remains intact. Thus, *Srr2* is dispensable for structural maintenance but essential for sustaining the transcriptional and epigenetic programs underlying lineage specification.

## Conclusions

Together, these findings identify *Srr2* as a critical enhancer linking *Sox2*-dependent transcriptional regulation with chromatin accessibility in adult NSCs. By sustaining SOX2 expression and preserving an open chromatin state during proliferation, *Srr2* enables the timely activation of neurogenic and gliogenic programs during lineage progression. Our study therefore provides a mechanistic framework for understanding how enhancer-mediated regulation of pioneer transcription factor dosage maintains the balance between stem cell maintenance and differentiation during adult neurogenesis. Further studies investigating how *Srr2* interacts with chromatin-modifying complexes and extracellular niche signals may help elucidate how dynamic enhancer activity regulates neural plasticity in ageing and disease.

## Methods

### Generation of *Sox2*^*tm1(Guide1/1:SRR2)RLB*^ mice

The *Sox2 *^*tm1(Guide1/1:SRR2)RLB*^ allele was generated using the CRISPR/Cas9 technology. Single guide RNAs (sgRNAs) were designed (www.crispr.mit.edu) to induce deletion of the mouse *Srr2* enhancer (81 bp), situated 4 kb downstream of *Sox2* (UCSC mm9 genome chr13: 34,552,926—34,553,007). Two pairs of guides were designed: pair 1 (G1_5’ + G1_3’) and pair 2 (G4_5’ + G5_3’) and independently injected with Cas9 mRNA as previously described [32] to produce two different mouse strains with the same *Srr2* deletion, in order to discriminate between the effects of the desired mutation, which would be common to both guides, and potential off-target effects of the guides which would be in contrast specific to a particular pair. Founders were genotyped for *Srr2* deletion by PCR and mosaicism estimated using the MiSeq system (Illumina). Two founders were chosen to establish two independent strains and bred to obtain *Srr2*^*del/del*^ mice for analyses. All mice were maintained on a C57BL/6 J genetic background and housed under specific pathogen-free (SPF) conditions in a controlled environment with a 12 h light/dark cycle and ad libitum access to food and water. Animals were euthanized using Schedule 1 (S1K) methods.

All animal experiments carried out were approved under the UK Animals (Scientific Procedures) Act 1986 and under the project licenses n. 80/2405 and PP8826065 and by the Francis Crick Animal Welfare and Ethical Review Body (AWERB).

### Adult NSC cultures and differentiation

NSC cultures were established from the subependymal zone of adult WT and *Srr2*-deleted (*Srr2*^*del/del*^) mice as previously described [33, 56]. As these cultures were directly derived from genetically defined mice, no additional cell line authentication was performed beyond animal genotyping. Cultures were routinely screened for mycoplasma contamination and tested negative. NSCs from wild-type and *Srr2*^*del/del*^ mice were grown in neurosphere growth medium containing 20 ng/ml epidermal growth factor (EGF) (Invitrogen, 53003–018) and 10 ng/ml basic fibroblast growth factor (bFGF) (Sigma, F0291). For differentiation assays, single neurosphere cells were plated at a density of 40,000 cells/cm^2^ on Matrigel®-coated coverslips and maintained for 2 days (2 DIV) in control medium containing FGF (PROLIF). Subsequently, mitogens were withdrawn to allow for differentiation, and the cells were cultured in medium without mitogens supplemented with 2% FBS. Differentiating (DIFF) cultures were then analyzed either 24 h (2 + 1 DIV) or 5 days (2 + 5 DIV) after mitogen removal (see Fig. 1A). 10 µM EdU was administered for 1 h prior to fixation.

### Immunofluorescence, image acquisition, and pre-processing

Immunofluorescence in vitro and in vivo was performed as previously described [57]. For in vivo experiments, mice were perfused with 4% w/v paraformaldehyde in phosphate-buffered saline (PBS), brains harvested, and cryosectioned at 50 μm. Sections were blocked with blocking solution (10% v/v donkey serum in PBS/0.1% v/v Triton X-100; PBST) for 1 h, then incubated with primary antibodies in 10% blocking solution overnight at 4 °C. The following primary antibodies were used for both in vitro and in vivo experiments: goat anti-SOX2 (Neuromics, 1:500), rabbit anti-SOX9 (Abcam AB184547, 1:1,000), mouse anti-ASCL1 (BD 556604, 1:500), rabbit anti-OLIG2 (Millipore AB9610, 1:500), rabbit anti-Ki67 (Abcam ab15580, 1:300), mouse anti-O4 (Hybridoma Bank rip, 1:300), rabbit anti-βIII-Tubulin (Sigma T2200, 1:300), rabbit anti-SOX10 (Roche Ventana, 1:500), rabbit anti-FOXG1 (Abcam Ab18259, 1:500), mouse anti-NeuN (Millipore MAB337, 1:500), chicken anti-MAP2 (Abcam AB5392, 1:500). EdU was developed with the Click-iT™ Plus EdU Alexa Fluor™ 555 Imaging Kit (ThermoFisher Scientific, C10638) following the manufacturer’s instructions. For in vivo tissue sections, samples were washed in PBST, then incubated for 1 h at room temperature with the corresponding anti-rat, anti-goat, or anti-rabbit secondary antibody conjugated to Alexa-Fluor 488, 555, 594 or 647 in 10% blocking solution with 1 μM 4’,6-diamino-2-phenylindole (DAPI). Sections were washed with PBST and mounted using Aqua-Poly/Mount (Polysciences, Inc., Warrington, PA, USA). For in vitro NSC cultures, images were acquired with an Olympus FV10i confocal microscope (Olympus), laser settings were first established on WT/control samples at the first time point (e.g., 2 DIV) and kept throughout the whole experiment. Random fields were imaged at the focal plane showing both the highest number of cells on focus and the most intense signal. 2 + 5 DIV differentiation samples were imaged using a Nikon ECLIPSE Ni-U microscope (Nikon) with a Zyla 4.2 sCMOS camera (Andor). Bioimage analysis was performed using the Fiji open-source software package [58]. ImageJ Macro Language scripts were developed to perform unbiased automatic analysis of the in vitro experiments. SOX2 was quantified with our previously developed “Cell proliferation HCS” tool [56]. A minimum of 500 cells per culture were analyzed in each condition. Cells were classified as Sox2^high^ using an intensity threshold of 400 arbitrary units (a.u.), determined from previous datasets and from the distribution of SOX2 signal intensities in WT and mutant samples. For in vivo experiments, laser settings were first established on WT tissue and similar regions of interest (ROI) were acquired in an Olympus FV10i confocal microscope. Maximal projection images were generated and the mean gray intensities of nuclear marker SOX2 were measured with Fiji software. Intensities were represented as frequency histograms normalized to the maximum count in each comparison. For in vivo tissue sections, images were acquired using a Leica SPE confocal microscope. Settings were established during the initial acquisition. All images taken from the Leica SPE confocal microscope were pre-processed using ImageJ (maximum z-projection) and the ones taken from VS120 Slide Scanner were processed using QuPath.

### RNA and ATAC seq

ATAC-seq and RNA-seq were performed as previously described [15]. Briefly, proliferating (2 DIV) and early differentiating (2 + 1 DIV) cells were harvested for RNA and chromatin extraction. For ATAC-seq, 50,000 cells were lysed, and nuclei were tagmented using the Nextera DNA Library Prep Kit (Illumina). Libraries were amplified (NEBNext) and sequenced on a HiSeq4000 platform to a depth of ~ 25 million reads/sample. For bulk RNA-seq, total RNA was extracted using QIAzol and the miRNeasy Micro Kit (Qiagen). cDNA synthesis and library preparation were performed with Tecan’s Ovation RNA-seq and Ultralow V2 kits. Libraries were sequenced under the same conditions. RNA and library quality were assessed using Agilent platforms.

### RNA sequencing

RNA-seq was performed as previously described [15]. Briefly, FASTQ files were preprocessed in the Francis Crick Institute, through RSEM package (v. 1.3.30) and STAR alignment (v. 2.5.2a). RSEM default parameters were maintained except for the forward-prob, which was established at 0.5. Differential expression analysis between *Srr2*^*del/del*^ and WT was performed with the default DESeq2 pipeline in R. Principal Component Analysis (PCA), hierarchical clusters, and heatmaps were evaluated to assess the quality of the samples and their replicates. An adjusted p-value smaller than 0.05 was set as the threshold for statistical significance. Volcano plots were generated in R to represent significantly up-regulated and down-regulated genes. The lists of differentially regulated genes were used as inputs for functional enrichment analysis. We used the compareCluster function as implemented in the clusterProfiler R package, with the following parameters: fun = “enrichGO”, OrgDb = “org.Mm.eg.db”, ont = ”BP”. Enriched GO term redundancy was minimized by using the simplify function of the same package, applying the following parameters: cutoff = 0.5, by = “p.adjust”, select_fun = min, measure = “Wang”, semData = NULL. Heatmaps were generated using the pheatmap package to display expression patterns across *Srr2*^*del/del*^ and WT samples. Prior to visualization, normalized counts were transformed using the regularized log (rlog) function of DESeq2, and the resulting values were row-wise scaled (z-score) to emphasize relative expression differences across samples.

### ATAC sequencing

ATAC-seq was performed as previously described [15]. Briefly, paired-end sequencing files were analyzed with FastQC (http://www.bioinformatics.babraham.ac.uk/projects/fastqc/), summarized with MultiQC, and visually inspected for major quality issues. We used Cutadapt to clip the Nextera 3ʹ R1 and R2 adapters and then Trimmomatic to trim low-quality portions and filter reads shorter than 20 bases. Then, a second round of quality control with FastQC and MultiQC was performed to make sure the outputs were in line with the expected results. High-quality reads were then mapped to the mouse genome with Bowtie2, using the *Mus musculus* GRCm38 (mm10) genome as reference and allowing mate dovetailing. Duplicates were marked on BAM files with MarkDuplicates from Picard tools (https://broadinstitute.github.io/picard/), insert sizes were calculated with the CollectInsertSizeMetrics tool from SAMtools. The biological replicates of each ATAC-seq sample were merged and used as input for ngs.plot to generate average metagene plots and per-gene accessibility signal heatmaps around TSS genomic coordinates of protein-coding genes. Peak calling was performed with MACS2, choosing narrow peaks, no model, and 0.05 False Discovery Rate (FDR) as options. The resulting narrow-peak BED files were manually loaded into the IGV genome browser alongside their corresponding ATAC-seq coverage tracks generated with the bamCoverage function from deepTools2 for visual inspection. Biological replicates of each sample were merged and used to generate average metagene plots and per-gene accessibility heatmaps around TSS genomic coordinates of mouse protein-coding genes with ngs.plot. MACS2 narrow-peak files and the corresponding BAM alignment files they were derived from were used as input for differential peak calling with DiffBind: https://bioconductor.org/packages/release/bioc/html/DiffBind.html. A false discovery rate threshold of 0.05 was chosen for peak differential detection between comparisons. Peaks that were significantly more or less concentrated in one sample compared to its control were considered open and closed, respectively. In order to associate both differential (open and closed) and non-differential peaks to their genomic contexts, we used ChIPseeker with the Gencode M18 mouse GTF annotation. Upstream and downstream regions of 3 Kb were allowed, as well as a flanking gene distance option of 5 Kb. We focused on peaks associated with 5ʹ and promoter regions, as determined by the ChIPseeker annotation, for downstream analysis. In the cases where there was more than one promoter peak associated with the same gene, we kept either the one that was closest to the TSS or the largest overlapping one.

Functional enrichment analysis of differentially accessible genes was carried out using STRING (https://string-db.org) and clusterProfiler. The Epigenetic Landscape In Silico deletion Analysis (Lisa) tool was used to discover associations of our target gene lists with regulatory TFs and chromatin factors based on the Cistrome DNase and ChIP-seq database (CistromeDB).

To assess whether the overlap between gene sets was greater than expected by chance, we calculated the expected overlap and statistical significance using a hypergeometric framework. The expected number of overlapping genes was computed as the product of the sizes of the two gene lists divided by the total number of genes in the genomic background, defined as all genes with detectable signal in the corresponding RNA-seq and ATAC-seq datasets. Enrichment was quantified using a Representation Factor, defined as the ratio between the observed overlap and the expected overlap. Statistical significance was evaluated using the hypergeometric distribution, testing the probability of observing the given overlap or greater under a random sampling model.

### Statistical analysis

Statistical analyses were performed using Prism v.8.0c (GraphPad Software, USA). To examine the significance of the data, tests were selected according to the experiment analyzed. When comparing two groups of values with normal distribution, unpaired t-test was performed. When comparing two groups of values where the distribution was nonparametric, Mann–Whitney test was performed. All results are represented as means ± standard deviation (SD) for raw data and means ± standard error of the mean (SEM) for graphs. Standard significance levels were used: *p < 0.05, **p < 0.01, ***p < 0.001, ****p < 0.0001. Enrichment analysis was performed through MetaCore Clarivate Analytics (https://portal.genego.com/) and the obtained results were represented through dotplots.

### Availability of data and materials

RNA-seq data generated in this study have been deposited in the Gene Expression Omnibus (GEO) under accession number GSE319463 [59] for *Srr2*^*del/del*^ samples and GSE196329 [60] for WT samples. ATAC-seq data have been deposited in GEO under accession number GSE319464 [61] for *Srr2*^*del/del*^ samples and GSE196329 [60] for WT samples. Fluorescence microscopy images used for quantification in Fig. 1, Fig. 4 and Fig. S1–S2 have been deposited in Zenodo (https://zenodo.org/records/20142987) [62].

## Supplementary Information

Additional file 1: Supplementary figures and legends. Contains Figs. S1–S2.

Additional file 2: Supplementary tables. Results from differential gene expression analysis, enrichment analysis, and differentially accessible regions. Contains Tables S1–S8.

Additional file 3: Supplementary figures. Results from STRING analysis. Contains Figs. S1–S2.

## Data Availability

No datasets were generated or analysed during the current study.
