# Supplementary material for: Srr2-dependent SOX2 levels govern the chromatin and transcriptional landscape of adult neural stem cell fate decisions in mouse
Source: Genome Biol. 2026 Jun 25;27:208. doi: 10.1186/s13059-026-04126-7 (PMC13307697; doi:10.1186/s13059-026-04126-7)
Supplement: Supplementary file 1 — Additional file 1: Supplementary figures and legends. Contains Figs. S1–S2. [file 13059_2026_4126_MOESM1_ESM.pdf]

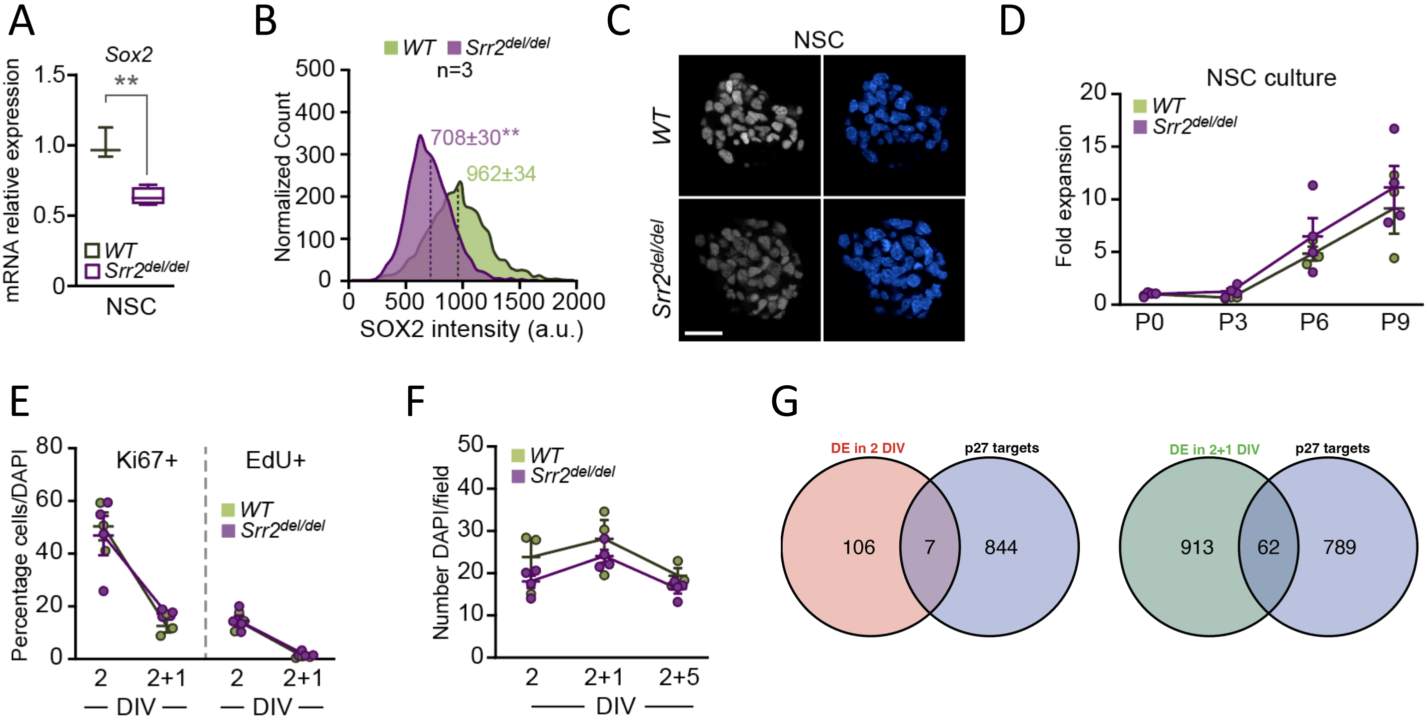

Fig. S1. *Srr2* deletion reduces SOX2 levels without affecting proliferation or cell survival in adult neural stem cell cultures. A) *Sox2* mRNA expression measured by RT–qPCR in neural stem cell (NSC) cultures derived from the SEZ of 2-month-old wild-type (WT) and *Srr2*$^{del/del}$ mice. **B)** Quantification of SOX2 protein levels by immunofluorescence (IF) in NSC cultures from WT and *Srr2*$^{del/del}$ mice. **C)** Representative immunocytochemistry images showing SOX2 expression in neurosphere cultures derived from the SEZ of 2-month-old WT and *Srr2*$^{del/del}$ mice. **D)** Growth curves showing the expansion of neurosphere cultures derived from WT and *Srr2*$^{del/del}$ adult neural stem cells across serial passages (P0, P3, P6, and P9) maintained in the presence of mitogens. **E)** Quantification of proliferative activity assessed by Ki67 immunostaining and EdU incorporation, expressed as the percentage of positive cells relative to total DAPI^{+} nuclei, in WT and *Srr2*$^{del/del}$ cultures at 2 days in vitro (2 DIV; proliferative expansion phase) and at 2+1 DIV (onset of differentiation). Bars represent mean ± SEM; each dot represents an independent culture. Two-way ANOVA revealed a significant effect of time but not genotype (Ki67: WT 2+1 DIV vs 2 DIV, *$p < 0.05$; *Srr2*$^{del/del}$ 2+1 DIV vs 2 DIV, *$p < 0.05$; EdU: WT 2+1 DIV vs 2 DIV, *$p < 0.05$; *Srr2*$^{del/del}$ 2+1 DIV vs 2 DIV, **$p < 0.01$). **F)** Quantification of total cell numbers in WT and *Srr2*$^{del/del}$ NSC cultures during proliferation (2 DIV) and early (2+1 DIV) and late (2+5 DIV) stages of differentiation. Bar graph shows the number of DAPI^{+} nuclei per field under each condition. No significant differences in total cell numbers were detected between genotypes at any stage analyzed, indicating the absence of overt cell loss during proliferation or differentiation. Bars represent mean ± SEM; each dot represents an independent culture. **G)** Venn diagrams showing the overlap between differentially expressed (DE) genes identified in *Srr2*$^{del/del}$ vs WT neural stem cells during proliferation (2 DIV) or early differentiation (2+1 DIV) and the p27-associated gene set reported by Biçer et al., 2017 [50], derived from p27 ChIP–seq peaks assigned to nearby genes. Only a limited overlap was observed in both conditions, with no significant enrichment of p27-associated targets among the DE genes (hypergeometric test; proliferation $p = 0.461$; 2+1 $p = 0.187$).

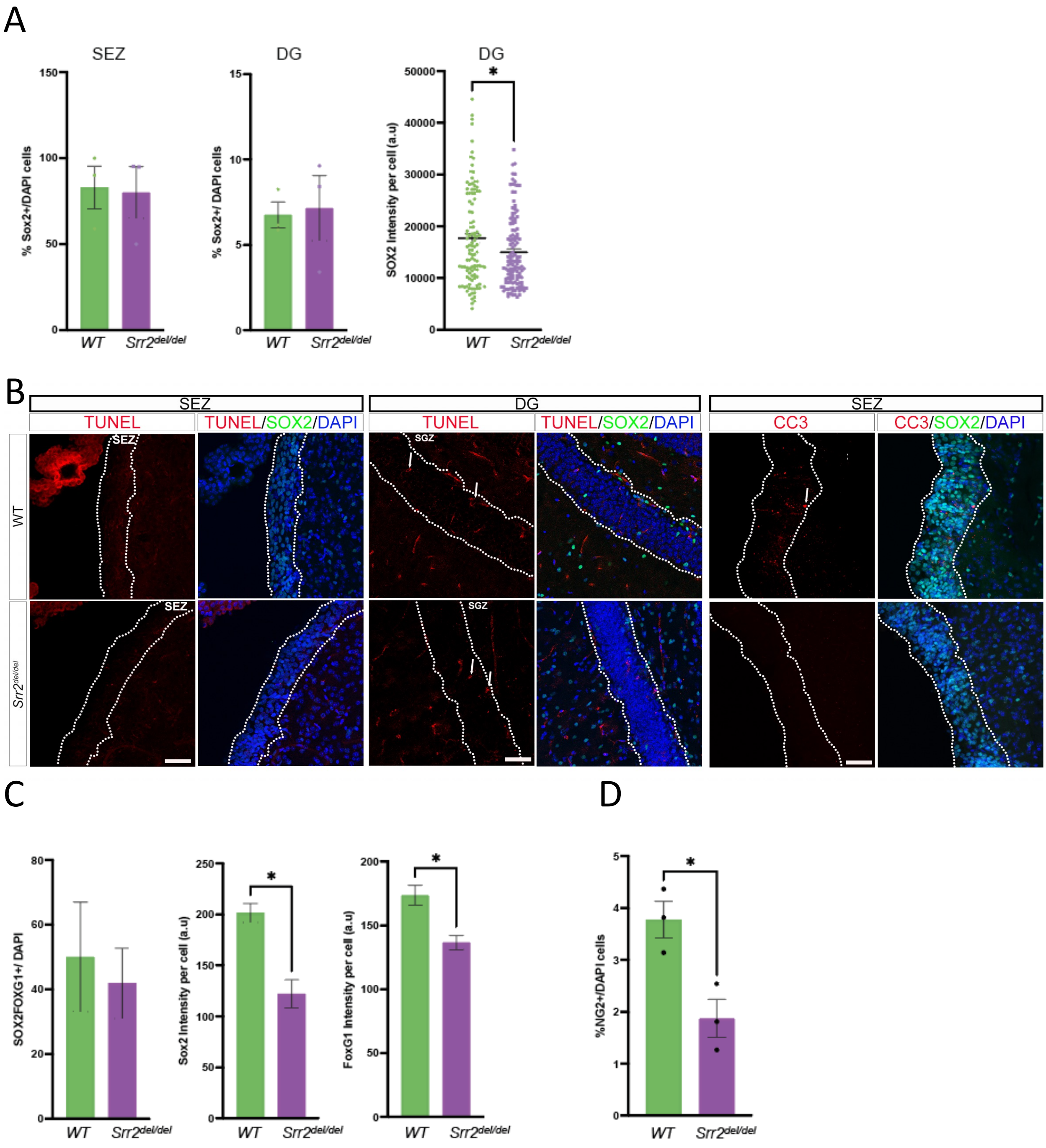

Fig. S2. *Srr2* deletion alters SOX2 expression and lineage-associated markers in adult neurogenic niches without inducing apoptosis. A) Quantification of the proportion of SOX2-positive cells relative to total DAPI$^+$ nuclei in the subependymal zone (SEZ; left panel) and dentate gyrus (DG; middle panel) of adult WT and *Srr2*$^{del/del}$ mice. Quantification of SOX2 fluorescence intensity at the single-cell level in the DG (right panel). Data are presented as mean ± SEM; each dot represents an individual cell. Statistical significance was assessed using Welch's t-test; *p < 0.05. **B)** Representative immunofluorescence images showing TUNEL (red) and SOX2 (green) staining in the SEZ and DG of adult WT and *Srr2*$^{del/del}$ mice. No differences between genotypes were observed. Right panels show immunostaining for cleaved Caspase-3 (CC3; red) and SOX2 (green) in the SEZ. CC3-positive cells were detected at low frequency and did not differ between genotypes. Nuclei are counterstained with DAPI (blue). Dashed lines delineate the neurogenic niches. Scale bars, 50 µm. **C)** Quantification of SOX2$^+$/FOXG1$^+$ cells in the SEZ of WT and *Srr2*$^{del/del}$ mice (left panel). Bar graph shows the percentage of SOX2$^+$/FOXG1$^+$ cells relative to total DAPI$^+$ nuclei within the SEZ. Quantification of SOX2 (middle panel) and FOXG1(right panel) fluorescence intensity, respectively, within SOX2$^+$/FOXG1$^+$ cells. Both SOX2 and FOXG1 signal intensities are significantly reduced in *Srr2*$^{del/del}$ brains. **D)** Quantification of NG2-positive cells in the SEZ of WT and *Srr2*$^{del/del}$ mice. Bar graph shows the percentage of NG2$^+$ cells relative to total DAPI$^+$ nuclei. *Srr2*$^{del/del}$ mice display a significant reduction in the proportion of NG2$^+$ cells compared with WT, indicating impaired oligodendroglial progenitor populations in vivo. Bars represent mean ± SEM; *p < 0.05 (unpaired two-tailed Student's t-test).
