# Supplementary material for: Srr2-dependent SOX2 levels govern the chromatin and transcriptional landscape of adult neural stem cell fate decisions in mouse
Source: Genome Biol. 2026 Jun 25;27:208. doi: 10.1186/s13059-026-04126-7 (PMC13307697; doi:10.1186/s13059-026-04126-7)
Supplement: Supplementary file 3 — Additional file 3: Supplementary figures. Results from STRING analysis. Contains Figs. S1–S2. [file 13059_2026_4126_MOESM3_ESM.pdf]

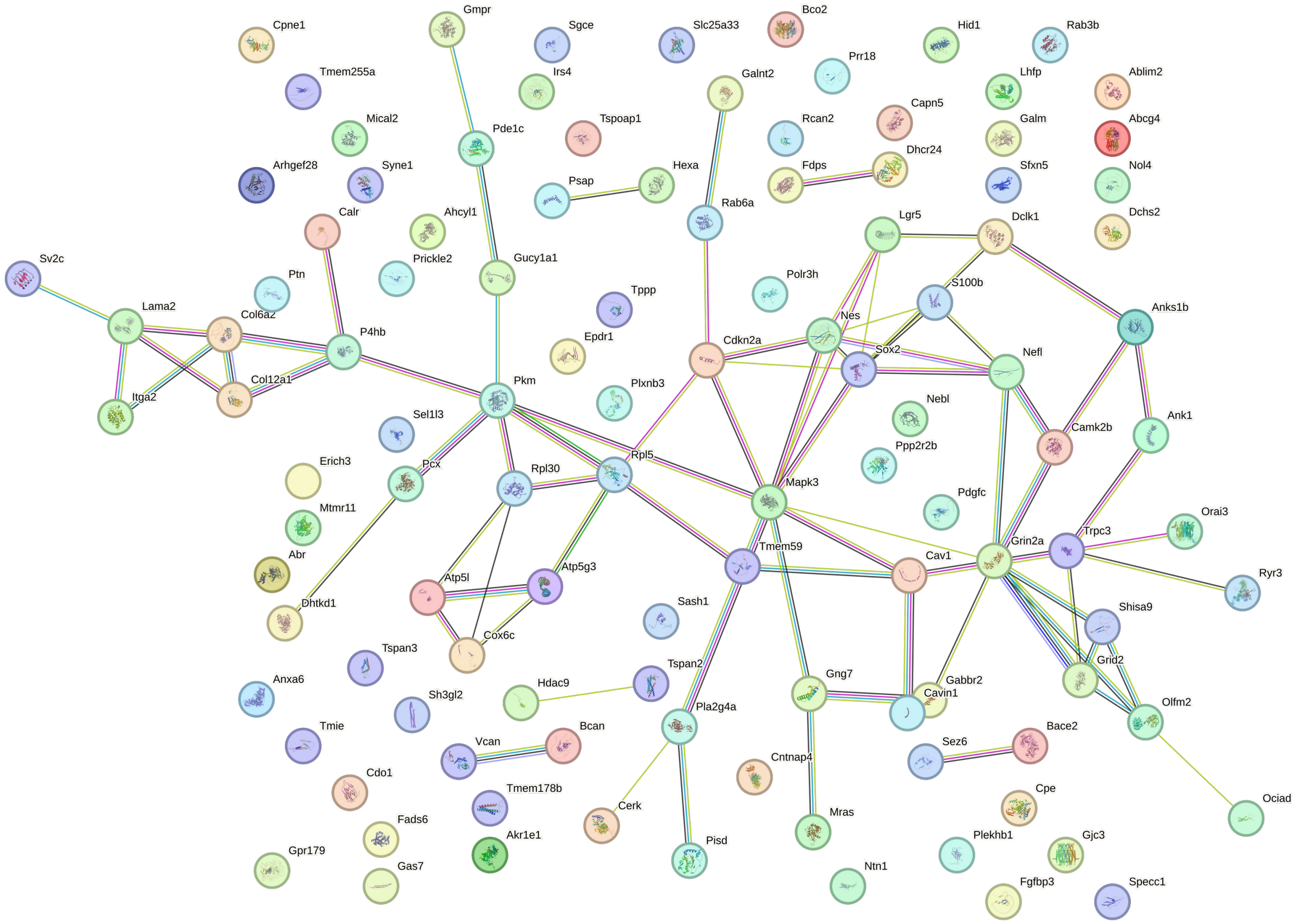

Fig. S1. Protein–protein interaction network generated from the complete set of 115 genes showing reduced transcriptional induction during early differentiation in mutant cells (STRING PPI enrichment p = 0.00505). The network was generated using STRING v12.0 (https://string-db.org/)

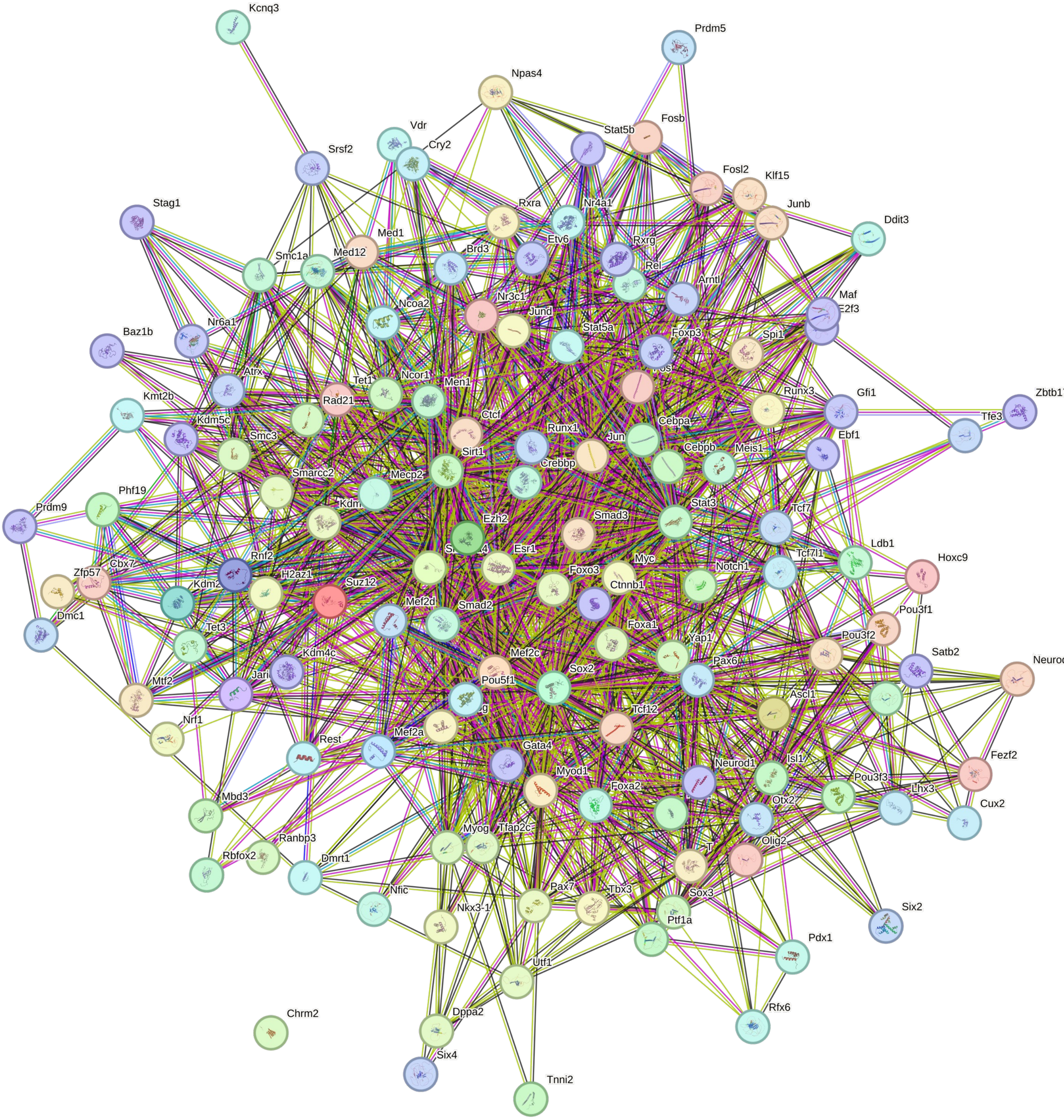

Fig. S2. Protein–protein interaction network generated from the complete set of 140 putative candidate transcription factors and chromatin regulators identified in the analysis. The network was generated using STRING v12.0 (https://string-db.org/).
